# Downregulation of CD40 expression contributes to the accumulation of myeloid-derived suppressor cells in gastric tumors

**DOI:** 10.3892/ol.2014.2174

**Published:** 2014-05-26

**Authors:** JIN SHEN, XIAOJUAN CHEN, ZHENXING WANG, GUANGBO ZHANG, WEICHANG CHEN

**Affiliations:** 1 Department of Gastroenterology, The First Affiliated Hospital of Soochow University, Suzhou, Jiangsu 215006, P.R. China; 2 Department of Oncology, The First Affiliated Hospital of Soochow University, Suzhou, Jiangsu 215006, P.R. China; 3 Key Laboratory of Medicine and Clinical Immunology of Jiangsu Province, The First Affiliated Hospital of Soochow University, Suzhou, Jiangsu 215006, P.R. China

**Keywords:** CD40, myeloid-derived suppressor cell accumulation, apoptosis

## Abstract

An elevated number of myeloid-derived suppressor cells (MDSCs) in tumor-bearing hosts has been recognized as a crucial mediator of tumor progression due to the cells potent ability to suppress antitumor immunity. Cluster of differentiation (CD) 40, as a suppressive phenotype expressed in MDSCs, is essential for MDSC-mediated immune suppression and the expansion of T regulatory cells. However, whether CD40 exerts a direct effect on the accumulation of MDSCs remains unclear. In the present study, CD40 was observed to be highly expressed on the MDSCs obtained from mice bearing gastric tumors. Notably, a significant decrease in the level of CD40 expression was observed in addition to an increased number of MDSCs during tumor progression. Further analysis revealed that the MDSC levels were found to positively correlate with tumor progression and that CD40 expression levels inversely correlate with the accumulation of MDSCs. To confirm the potent correlation between CD40 expression and the accumulation of MDSCs, the apoptosis of the MDSCs was detected using agonistic anti-CD40 treatment. The results indicated that CD40 activation induces apoptosis in MDSCs and that the downregulation of CD40 expression may contribute to MDSC accumulation by facilitating MDSC resistance to apoptosis. Thus, these observations provide a novel mechanism to improve our understanding of the involvement of CD40 in MDSC accumulation during cancer development.

## Introduction

Myeloid-derived suppressor cells (MDSCs) represent a heterogeneous population of immature myeloid cells that are characterized by their potential to suppress various T cell functions (1). MDSCs are enriched in tumor-bearing hosts, and since the accumulation of MDSCs has been confirmed to be correlated with tumor progression (2), the control of MDSC accumulation is crucial for directly targeting MDSCs against tumors. During tumor development, tumor- and host-secreted factors, including vascular endothelial growth factor, stem cell factor, granulocyte-macrophage colony-stimulating factor, interleukin (IL)-1β, IL-6 and prostaglandin E2, have been reported to promote the accumulation of MDSCs. These factors can expand and differentiate MDSCs by interacting with the receptors on their surface causing conversion to a suppressive phenotype (1,3). Certain molecules that are expressed on the surface of MDSCs regulate the accumulation or suppressive activity of MDSCs, including cluster of differentiation (CD) 80 (4), CD115 (5), IL-4 receptor α (IL-4Rα) (6), programmed cell death ligand-1 (7), CD40 (8) and CD95 (Fas) (9).

CD40, a member of the tumor necrosis factor (TNF) receptor superfamily, is known to be expressed at various levels on antigen-presenting cells, epithelial cells, hematopoietic progenitor cells and activated T cells (10,11). The interaction between the CD40-CD40 ligand has been demonstrated to regulate immune responses, and CD40 has an important effect on promoting tumor cell apoptosis or tumor growth. The dual role of CD40 depends on the level of its expression (12) and different signaling activation (24). Recent studies have shown that the expression of CD40 on MDSCs regulates MDSC-mediated immune suppression and the expansion of T regulatory cells (Tregs) (8,13,14). However, whether CD40 is a mediator of MDSC accumulation has not yet been thoroughly investigated.

Therefore, the present study evaluated CD40 expression on the MDSCs of a gastric tumor model, with a focus on the dynamics of tumor progression and CD40 expression on MDSCs. The levels of CD40 expression were observed to significantly correlate with MDSC accumulation and the relative contribution to MDSC apoptosis. The present study aimed to analyze the regulation of MDSC levels in order to determine a method for targeting MDSCs against tumors.

## Materials and methods

### Cell line and tumor model

The mouse forestomach carcinoma (MFC) cell line was obtained from the Shanghai Institute of Biochemistry and Cell Biology, Shanghai Institute for Biological Sciences (Shanghai, China) and 6–10 week old C57BL/6 (B6) mice were purchased from the Shanghai Laboratory Animal Center, Chinese Academy of Sciences (Shanghai, China). All mice were housed in pathogen-free conditions at the Animal Center of the Medical College of Soochow University (Suzhou, China) according to the institutional guidelines for animal care and use. The cells were cultured in Roswell Park Memorial Institute 1640 medium (Gibco-BRL, Carlsbad, CA, USA) containing 10% fetal calf serum. A total of 1×10^6^ MFC cells were then subcutaneously injected into the B6 mice and the tumor sizes were evaluated every two to three days. The study was approved by the ethics committee of Soochow University (Suzhou, China).

### Antibodies and flow cytometry

Phycoerythrin-cyanine **(**PC)7-labeled anti-Gr-1 monoclonal antibody (mAb), PC5-labeled anti-CD11b mAb, phycoerythrin (PE)-labeled anti-CD40 mAb and agonistic anti-CD40 antibody were provided by Biolegend (San Diego, CA, USA). The spleens and primary tumors were harvested at varying tumor sizes, placed in phosphate-buffered saline (PBS), gently pressed and then filtered to obtain a single cell suspension. Following red blood cell lysis and washing with PBS, the splenocytes and tumor tissue cells were then stained with the aforementioned mouse-specific mAbs for 30 min at 4°C. All samples were analyzed by flow cytometry (Cytomics FC 500, Beckman Coulter, Miami, FL, USA).

### MDSC apoptosis

Single cell suspensions (2×10^6^ cells/ml) were prepared from the spleen and tumor tissues and incubated with PBS or agonistic anti-CD40 (5 μg/ml; Biolegend) for 24 h at 37°C in a humidified atmosphere of 5% CO_2_. The cells were subsequently collected and stained with anti-Gr-1 mAb, anti-CD11b mAb and Annexin V (Invitrogen Life Technologies, Carlsbad, CA, USA), as described previously (20). Finally, the proportion of the Gr-1^+^/CD11b^+^/Annexin V^+^ cells was determined by flow cytometric analysis.

### Statistical analysis

Statistical analysis of the data was performed using FlowJo software (TreeStar Inc., Ashland, OR, USA), and all statistical analyses were performed using GraphPad Prism software (GraphPad Software, Inc., La Jolla, CA, USA). The statistical differences were analyzed using a t-test and one-way or two-way analysis of variance. The correlation between tumor progression and the expression of CD40 on the MDSCs, as determined by fluorescence-activated cell sorting, was assessed by Pearson’s correlation analysis. Data corresponding to the expression of CD40 and MDSC apoptosis are presented as the mean ± standard error of the mean (SEM). P<0.05 was considered to indicate a statistically significant difference and all P-values were two-sided.

## Results

### CD40 expression on MDSCs of gastric tumor-bearing mice

**Figure 1:**
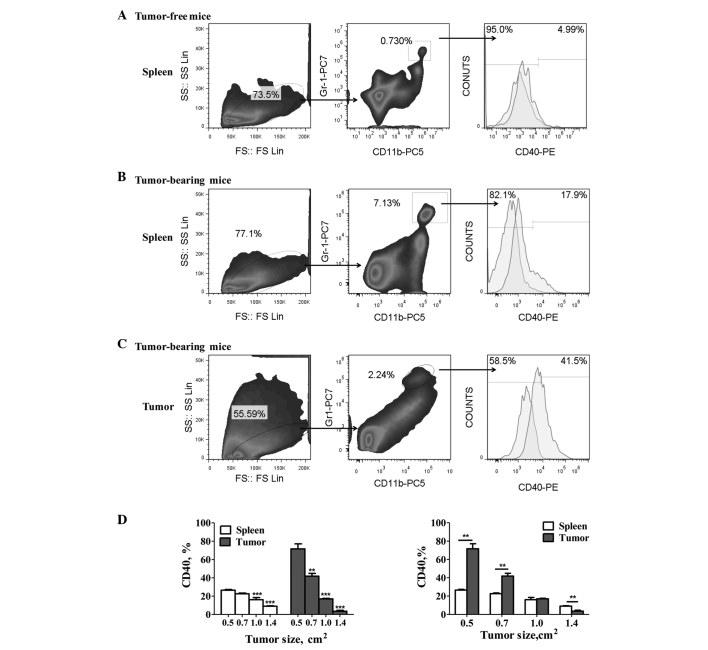
CD40 expression on Gr-1^+^/CD11b^+^ MDSCs. Splenocytes obtained from (A) a tumor-free mouse and (B) mice bearing MFC gastric tumors were stained with anti-Gr-1, anti-CD11b and anti-CD40. Data are presented as the percentage of CD40^+^ MDSCs in gated Gr-1^+^/CD11b^+^ MDSCs. (C) Percentage of CD40^+^ MDSCs from MFC gastric tumor tissues cells. (D) CD40 expression in MDSCs from MFC gastric tumor tissues and splenocytes was analyzed at different tumor sizes during tumor progression. Each experiment was performed in triplicate and data are presented as the mean ± standard error of the mean. ^*^P<0.05, ^**^P<0.01 and ^***^P<0.0001 vs. control. MDSCs, myeloid-derived suppressor cells; MFC, mouse forestomach carcinoma; PE, phycoerythrin; PC, phycoerythrin-cyanine; CD, cluster of differentiation; FS, forward scatter; SS, side scatter.

The levels of CD40 expression were detected on the Gr-1^+^/CD11b^+^ MDSCs from gastric tumor-bearing and tumor-free mice. The results showed a significant increase in the percentage of CD40^+^ MDSCs in the tumor-bearing mice compared with the tumor-free mice (Fig. 1A and B). Furthermore, tumor-infiltrating MDSCs exhibited significantly higher CD40 expression levels than the spleen-derived MDSCs (P<0.05; Fig. 1B and C). However, significant downregulation of CD40 expression on the MDSCs was observed with tumor progression (P<0.05; Fig. 1D). In addition, the levels of CD40 expression on the tumor-derived MDSCs decreased gradually from 71.67±5.42 to 3.56±0.99% (mean ± SEM), and that of the splenic MDSCs decreased from 26.53±0.82 to 9.05±0.43% (mean ± SEM).

### Correlation between CD40 downregulation and MDSC accumulation with tumor progression

**Figure 2:**
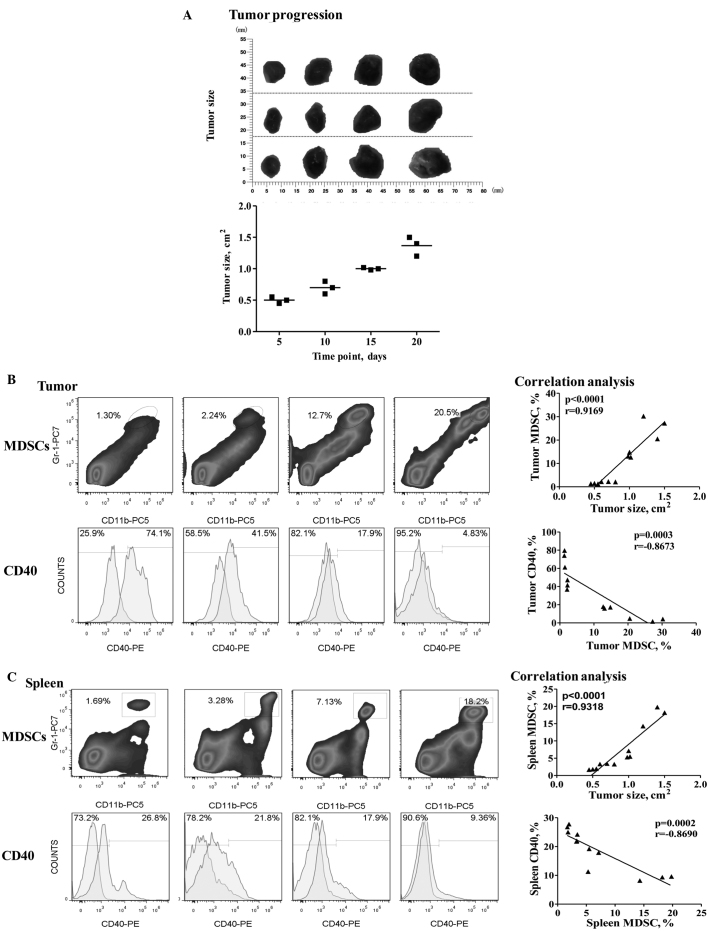
CD40 downregulation correlates with MDSC accumulation during tumor progression. (A) Primary tumor growth was evaluated by tumor size (tumor length and width) every two to three days. The results of three independent experiments are shown. (B) CD40 expression on tumor-infiltrating MDSCs was observed with tumor progression. Representative examples are shown from three independent experiments. The correlation between MDSC accumulation and tumor progression or CD40 expression was assessed using Pearson’s correlation analysis. (C) Similar results were obtained from splenocytes. MDSCs, myeloid-derived suppressor cells; PE, phycoerythrin; PC, phycoerythrin-cyanine; CD, cluster of differentiation.

In gastric tumor-bearing mice, tumor burden was found to induce MDSC accumulation. The proportion of MDSCs in primary tumors increased from 1.38±0.05 to 25.97±2.87% (mean ± SEM) and similarly, the proportion of MDSCs in the spleen increased from 1.82±0.08 to 17.43±1.63% (mean ± SEM). However, CD40 expression in the MDSCs was significantly downregulated, as aforementioned. The correlation analysis revealed a significant positive correlation between the number of tumor-infiltrating MDSCs and tumor progression (r=0.9169; P<0.0001; Fig. 2B), which was also observed between the splenic MDSCs and tumor progression (r=0.9318; P<0.0001; Fig. 2C). Furthermore, the CD40 expression levels were found to exhibit a significant inverse correlation with MDSC accumulation in the tumors (r=−0.8673; P=0.0003; Fig. 2B), which was also observed in the spleen (r=−0.8690; P=0.002; Fig. 2C).

### Regulation of MDSC accumulation through CD40-induced apoptosis in vivo

**Figure 3:**
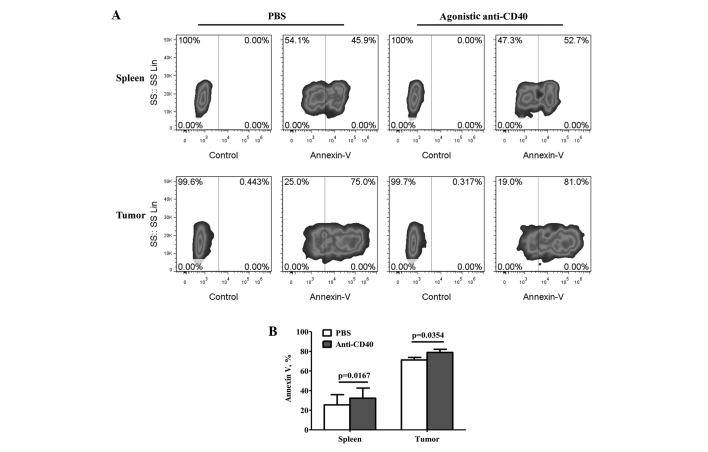
CD40-induced apoptosis regulates MDSC accumulation. (A) Splenocytes and tumor tissue cells obtained from tumor-bearing mice were incubated with PBS or agonistic anti-CD40 for 24 h and stained with anti-Gr-1, anti-CD11b and Annexin V. Data are presented as the percentage of Annexin V^+^ cells in gated Gr-1^+^/CD11b^+^ MDSCs. (B) Proportion of Annexin V^+^/Gr-1^+^/CD11b^+^ cells from tumors and spleens expressed as the percentage of total Gr-1^+^/CD11b^+^ cells. Data are presented as the mean ± standard error of the mean (n=3 per group). ^*^P<0.05 vs. control. MDSCs, myeloid-derived suppressor cells; PBS, phopshate-buffered saline; CD, cluster of differentiation; FS, forward scatter; SS, side scatter.

To further investigate the correlation between the CD40 expression levels and MDSC accumulation during tumor growth, the apoptosis of the MDSCs was detected by treatment with agonistic anti-CD40 or PBS. The apoptosis of the MDSCs was found to significantly increase following treatment with agonistic anti-CD40 (Fig. 3B), and compared with the PBS treatment, the percentage of apoptotic tumor-infiltrating MDSCs increased from 71.37±2.68 to 79.09±3.29% (mean ± SEM; P=0.0352; Fig. 3A and B). Furthermore, the percentage of apoptotic splenic MDSCs increased from 25.43±10.45 to 32.30±10.27% (mean ± SEM; P=0.0167; Fig. 3A and B).

## Discussion

MDSCs contribute to tumor progression via their potent ability to suppress antitumor immunity and induce tumor escape. Limiting MDSC accumulation using gene ablation, drugs or antibodies enhances antitumor immunity and may result in tumor regression (15–18). Thus, regulating MDSC accumulation is a powerful tool to inhibit tumor progression. Previous studies have reported the expression of CD40 on MDSCs to be important in MDSC-mediated immune suppression and Treg expansion in certain tumor models (8,13,14). However, few studies have analyzed the direct effects of CD40 expression on MDSCs, including differentiation and expansion. Weiss *et al* (19) revealed that the combination of IL-2 with agonistic anti-CD40 reduces the number of MDSCs in the tumor microenvironment by mediating MDSC recruitment. Furthermore, Zhao *et al* (20) showed that TNF signaling mediates MDSC accumulation by affecting their apoptosis rather than proliferation. However, whether CD40 regulates MDSC accumulation via apoptosis has not yet been investigated.

The results of the current study showed that CD40 is highly expressed on the MDSCs of a gastric tumor model. The tumors were found to induce MDSC accumulation, and the MDSCs exhibited different levels of CD40 expression with tumor progression. CD40 was preferentially expressed on tumor-infiltrating MDSCs at the early phase of progression, but was gradually downregulated with tumor progression. These differences may be attributed to the diverse tumor types or the various sites (including the spleen, tumor and blood) and progression stages.

Since tumor progression affects the differentiation of MDSCs towards a suppressive phenotype, the downregulation of CD40 may correlate with the functional state of the MDSCs (21). Notably, a marked correlation was identified among MDSC accumulation, CD40 expression and tumor progression. The downregulation of CD40 was found to significantly correlate with MDSC accumulation, which indicated that CD40 may be involved in MDSC accumulation. Considering that CD40 has a dual role in the regulation of cell apoptosis, which relies on CD40 expression levels, whereby high levels of CD40 expression induce apoptosis and low levels prevent apoptosis, we hypothesized that MDSC accumulation may be regulated by CD40 via apoptosis. The results of the present study revealed that treatment with agonistic anti-CD40 significantly increases MDSC apoptosis, which indicates that CD40 activation induces MDSC apoptosis. Therefore, the downregulation of CD40 expression may facilitate MDSC resistance to apoptosis and thereby promote the accumulation of the MDSCs. Furthermore, the present study showed that treatment with agonistic anti-CD40 was effective in promoting the apoptosis of MDSCs and inhibiting MDSC accumulation.

The mechanisms underlying CD40-mediated MDSC apoptosis remain unclear. To date, studies have reported that Fas signaling mediates MDSC apoptosis (9,22). In addition, inhibition of IL-4Rα/signal transducer and activator of transcription (STAT)-6 (15) or knockout of TNF signaling enhances MDSC apoptosis (20) and subsequently results in tumor regression. CD40, as a member of the TNF superfamily, may use similar signaling pathways as TNF to regulate MDSC apoptosis. In addition, CD40 has been demonstrated to interact with Fas to mediate cell apoptosis (23). Furthermore, CD40 downstream signaling molecules, including activator protein 1 (c-Jun and c-Fos) and STAT-3, are also associated with cell apoptosis (24,25). Therefore, the possible mechanisms of CD40-mediated MDSC apoptosis remain to be elucidated. Further studies are required to investigate whether these factors function separately, in a synergistic manner or via an interaction with CD40 to regulate MDSC accumulation.

In conclusion, immunotherapeutic interference with MDSC accumulation and its functions present a potential strategy for tumor treatment. In addition, CD40 is the most promising remedial molecular target against tumors. The current study demonstrated that CD40 mediates the accumulation of MDSCs via the induction of MDSC apoptosis, therefore, CD40 may present a novel target for decreasing MDSC levels in the tumor environment.

## Abbreviations

MDSCs: myeloid-derived suppressor cells
Tregs: T regulatory cells
MFC: mouse forestomach carcinoma
mAb: monoclonal antibody
